# Functional differentiation of midbrain neurons from human cord blood-derived induced pluripotent stem cells

**DOI:** 10.1186/scrt423

**Published:** 2014-03-17

**Authors:** Nancy Stanslowsky, Alexandra Haase, Ulrich Martin, Maximilian Naujock, Andreas Leffler, Reinhard Dengler, Florian Wegner

**Affiliations:** 1 Department of Neurology, Hannover Medical School, Carl-Neuberg-Str. 1, 30625 Hannover, Germany; 2 Center for Systems Neuroscience, Hannover, Germany; 3 Department of Cardiac, Thoracic, Transplantation, and Vascular Surgery, Leibnitz Research Laboratories for Biotechnology and Artificial Organs (LEBAO), Hannover Medical School, Hannover, Germany; 4 REBIRTH-Cluster of Excellence, Hannover, Germany; 5 Department of Anaesthesia and Critical Care Medicine, Hannover Medical School, Hannover, Germany

## Abstract

**Introduction:**

Human induced pluripotent stem cells (hiPSCs) offer great promise for regenerative therapies or *in vitro* modelling of neurodegenerative disorders like Parkinson’s disease. Currently, widely used cell sources for the generation of hiPSCs are somatic cells obtained from aged individuals. However, a critical issue concerning the potential clinical use of these iPSCs is mutations that accumulate over lifetime and are transferred onto iPSCs during reprogramming which may influence the functionality of cells differentiated from them. The aim of our study was to establish a differentiation strategy to efficiently generate neurons including dopaminergic cells from human cord blood-derived iPSCs (hCBiPSCs) as a juvenescent cell source and prove their functional maturation *in vitro*.

**Methods:**

The differentiation of hCBiPSCs was initiated by inhibition of transforming growth factor-β and bone morphogenetic protein signaling using the small molecules dorsomorphin and SB 431542 before final maturation was carried out. hCBiPSCs and differentiated neurons were characterized by immunocytochemistry and quantitative real time-polymerase chain reaction. Since functional investigations of hCBiPSC-derived neurons are indispensable prior to clinical applications, we performed detailed analysis of essential ion channel properties using whole-cell patch-clamp recordings and calcium imaging.

**Results:**

A Sox1 and Pax6 positive neuronal progenitor cell population was efficiently induced from hCBiPSCs using a newly established differentiation protocol. Neuronal progenitor cells could be further maturated into dopaminergic neurons expressing tyrosine hydroxylase, the dopamine transporter and engrailed 1. Differentiated hCBiPSCs exhibited voltage-gated ion currents, were able to fire action potentials and displayed synaptic activity indicating synapse formation. Application of the neurotransmitters GABA, glutamate and acetylcholine induced depolarizing calcium signal changes in neuronal cells providing evidence for the excitatory effects of these ligand-gated ion channels during maturation *in vitro*.

**Conclusions:**

This study demonstrates for the first time that hCBiPSCs can be used as a juvenescent cell source to generate a large number of functional neurons including dopaminergic cells which may serve for the development of novel regenerative treatment strategies.

## Introduction

Human induced pluripotent stem cells (hiPSCs) derived from somatic cells hold great promise to study and treat neurodegenerative diseases. IPSCs are self-renewing and able to differentiate into neurons similarly to human embryonic stem cells (hESCs), but without the negative ethical connotation [1-3]. hiPSCs offer the advantage of providing an unlimited source of genetically personalized cells with a diminished risk of immunorejection, which seems attractive for regenerative cell therapies, such as the replacement of dopaminergic (DA) neurons in Parkinson’s disease [4-11]. Even though the functionality of neuronal cells generated *in vitro* is of high relevance for preclinical and clinical studies, investigation of the functional properties of hiPSCs-derived neurons is rare [12-14].

In various studies somatic cells or stem cells from adult individuals were used to generate hiPSCs [15-19]. The usage of juvenile rather than aged human cells for generation of iPSCs is expected to have the advantage of lacking genetic mutations that tend to accumulate in adult stem and somatic cells over a lifetime, contributing to aging processes and cancer formation [20-22]. Although epigenetic reprogramming occurs and telomerase activity is restored during the process of pluripotency induction [23,24], genomic and chromosomal abnormalities acquired in aged cells are not rectified and may influence the functionality of cells differentiated from those iPSCs. Besides their juvenile character, the utilization of human cord blood endothelial cells for the generation of iPSCs has further advantages. They can be easily collected without invasive procedures and the emergence of public and commercial cord blood banks predestines them for future clinical applications.

Over the last decade, various tissue culture protocols have emerged that recapitulate the DA differentiation process in hESCs and hiPSCs. Some approaches focused on stromal feeder cell co-cultures to promote DA differentiation [9,11,25-35], others simply withdrew mitogens crucial for the maintenance of pluripotency to induce neuronal differentiation [6,10,36-46]. Stromal feeder cells have the disadvantage of introducing greater variability in the differentiation process by secreting undefined factors. Although media components are defined in differentiation concepts in which mitogens were withdrawn, the signaling cascades leading to neural induction are not fully understood yet. Recently, the utilization of small molecule inhibitors of transforming growth factor-β (TGF-β) and bone morphogenetic protein (BMP) signaling in the differentiation process became more popular because they induce neural conversion in a defined manner and have been shown to enhance neural conversion efficiency by inhibiting mesenchymal differentiation [5,31,39,47-51]. In general, TGF-β/BMP ligands initiate signaling by phosphorylation of cytoplasmatic SMAD proteins upon receptor binding. Activated SMADs translocate to the nucleus where they regulate the transcription of target genes. The molecules dorsomorphin (DM) and SB 431542 (SB) antagonize the TGF-β and BMP pathways and, therefore, affect biological processes including neuronal patterning [52].

In this study, we show for the first time that iPSCs generated from human cord blood-derived endothelial cells by means of lentiviral overexpression of the four factors OCT4, SOX2, LIN28 and NANOG as described by Haase *et al*. [53] are able to differentiate into DA neurons by dual SMAD inhibition and exhibit functional neuronal properties. The differentiated neurons revealed large voltage-gated currents and were able to fire action potentials (APs). Spontaneous synaptic activity indicated the formation of synaptic connections. We demonstrated neurotransmitter-induced calcium transients providing evidence for ligand-gated receptor expression and excitatory GABA actions during maturation *in vitro*. These data suggest that human cord blood-derived iPSC (hCBiPSC)-derived neurons including dopaminergic cells develop essential functional properties and may deliver a juvenescent source for novel regenerative cell therapies.

## Materials and methods

The use of human iPSCs in this study was approved by the local ethics committee of Hannover Medical School (Date: 08.07.2010, No. 776).

### Human iPSC culture and *in vitro* differentiation

The hCBiPSC lines were generated and characterized by Haase *et al*. [53]. In brief, all cord blood endothelial cell isolates showed high expression of endothelial markers including CD31 and CD146 [53]. Lentiviral transductions with *OCT4, SOX2, NANOG* and *LIN28* lead to reprogramming efficiencies of cord blood endothelial cells between 0.0001% and 0.03% [53]. The transplantation of undifferentiated hCBiPSCs into immunodeficient SCID-beige mice led to the formation of typical teratomas containing derivatives of all three germ layers [53]. Karyotype analyses revealed no abnormalities in the hCBiPSC clones [53].

Human CBiPSCs were expanded as described previously [53]. Undifferentiated hCBiPSCs were maintained on a feeder layer of mouse embryonic fibroblasts inactivated by gamma-irradiation (60 gray). Feeders were seeded at 1 × 10^5^ cells/well in a six-well plate (Nunc, Langenselbold, Germany) coated with 1% gelatin (Sigma-Aldrich, Taufkirchen, Germany). If not otherwise stated all media and medium supplements were purchased from Life Technologies (Darmstadt, Germany). Cytokines were obtained from Peprotech (Hamburg, Germany).

For differentiation, hCBiPSC colonies were detached from the feeder layer and cultured in suspension as embryoid bodies (EBs) for four days in knockout medium containing knockout DMEM, 20% knockout serum replacement, 0.1 mM MEM nonessential amino acids, 2 mM glutamax and 0.1 mM β-mercaptoethanol supplemented with 10 μM SB 431542 (SB, Biomol, Hamburg, Germany) and 1 μM DM (R&D Systems, Wiesbaden-Nordenstadt, Germany). On day four, the medium was changed to DMEM/F12 consisting of glutamax, N2 supplement, 10 μM SB, 1 μM DM, 0.6 μM purmorphamine (PMA, Biomol) and 100 ng/ml fibroblast growth factor 8 (FGF8). After six days, SB and DM were withdrawn. After six additional days in suspension, EBs were attached to poly-L-ornithine (20 μg/ml)/laminin (10 μg/ml)-coated cell culture plates and cultured in Neurobasal medium supplemented with glutamax, N2, B27 minus AO, 20 ng/ml BDNF, 20 ng/ml GDNF, 25 ng/ml TGFβ3, 200 μM ascorbic acid (Sigma-Aldrich) and 1 mM cAMP (Sigma-Aldrich) for up to 30 days.

### Immunocytochemistry

Cells were fixed in 4% paraformaldehyde and treated with blocking buffer (5% goat serum, 1% BSA, 0.3% Triton X-100 in PBS) for 45 minutes. Primary antibodies were applied overnight at 4°C. For visualization the appropriate fluorescence-labeled secondary antibodies were added for one hour at room temperature and nuclei were counterstained with 4’,6-diamidino-2-phenylindole (DAPI, 10 mg/ml, Life Technologies). The following primary antibodies were used: rabbit polyclonal anti-Oct4 (1:500, Cell Signaling, Danvers, Massachusetts, USA), mouse monoclonal anti-Pax6 (1:500, Millipore, Schwalbach, Germany), rabbit monoclonal anti-FoxA2 (1:250, Cell Signaling), mouse monoclonal anti-beta III tubulin (Tuj1, 1:500, Millipore), rabbit polyclonal anti-MAP2 (1:500, Millipore), rabbit polyclonal anti-TH (1:500, Santa Cruz Biotechnology, Heidelberg, Germany), rabbit polyclonal anti-GABA (1:1000, Sigma-Aldrich) and rabbit polyclonal anti-GFAP (1:500, Dako, Hamburg, Germany). Secondary antibodies were Alexa Fluor goat anti-mouse or anti-rabbit 488 or 555 (1:500, Life Technologies). All secondary antibodies were tested for specificity and cross reactivity.

Immunostainings were visualized by fluorescence microscopy (BX61; Olympus). Digital images were acquired with an Olympus DP72 camera using the image-analysis software Cell^F^ (Olympus). The number of cells immunoreactive for Tuj1, MAP2, TH, GABA or GFAP was determined related to the number of DAPI stained nuclei from at least three independent differentiation experiments. Approximately 1,000 cells were counted within three randomly selected visual fields.

### Reverse transcription and quantitative real-time PCR

Total RNA was extracted using the RNeasy kit (Qiagen, Hilden, Germany) and treated with DNase I according to the manufacturers’ instructions. For each reaction, 1 μg of total RNA was reversely transcribed using oligo-dT primer and Superscript II reverse transcriptase (Life Technologies).

Quantitative real-time PCR experiments were performed with cDNA from 50 ng total RNA, 1.75 μM forward and reverse primer and Power SYBR-Green PCR Master Mix (Life Technologies) in a StepOnePlus instrument (Applied Biosystems, Darmstadt, Germany) under the following amplification conditions: 95°C for 10 minutes, followed by 40 cycles of 95°C for 15 seconds and 60°C for 1 minute. The specificity of the PCR products was ensured by melting curve analysis. The correct amplicon size was confirmed by agarose gel electrophoresis using a low molecular weight DNA ladder (New England Biolabs, Ipswich, MA, USA; see Additional file 1: Figure S1). Equal PCR efficiency of all primer pairs was validated by serial cDNA dilution. For primer information see Additional file 2: Table S1.

For the quantification of target gene expression the threshold cycle (Ct) values of the targets were normalized against that of the endogenous reference β2-microglobulin (Ct (target) – Ct (reference) = ΔCt). ΔCt values were plotted as relative levels of gene expression and are given as means ± standard error of the mean (SEM) from three differentiation experiments.

### Electrophysiology

Patch pipettes were formed from borosilicate glass (Science Products, Hofheim, Germany) with a DMZ-universal puller (Zeitz-Instruments, Martinsried, Germany) and fire-polished to final resistances of 3 to 4 MΩ when filled with the internal solution consisting of 153 mM KCl, 1 mM MgCl2, 10 mM HEPES, 5 mM EGTA and 2 mM Mg-ATP, adjusted to pH 7.3 with KOH (305 mOsm). The bath solution contained 142 mM NaCl, 8 mM KCl, 1 mM CaCl2, 6 mM MgCl2, 10 mM glucose and 10 mM HEPES, adjusted to pH 7.4 with NaOH (325 mOsm). Tetrodotoxin (TTX, 1 μM), tetraethylammonium chloride (TEA, 10 mM), bicuculline (BIC, 10 μM) and 2,3-dihydroxy-6-nitro-7-sulphamoyl-benzo(f)quinoxaline (NBQX, 10 μM, all purchased from Sigma-Aldrich) were diluted in the bath solution and applied via gravity using a SF-77B perfusion fast-step system (Warner Instruments, Hamden, Connecticut, USA) as described previously [54]. The stock solution of BIC was dissolved in an external solution containing dimethyl sulfoxide (DMSO) at a maximal final concentration of 0.1%.

Whole-cell patch clamp experiments were performed at 20°C to 22°C under optical control (inverted microscope, Zeiss, Jena, Germany). Cells with leak currents <100 pA were used for further analysis. Whole-cell currents were low-pass filtered at 2.9 kHz, digitized at 10 kHz using an EPC-10 amplifier (HEKA, Lambrecht, Germany) and analyzed with Patch Master (HEKA).

### Calcium imaging

Monitoring of cytosolic calcium transients in individual neurons was carried out using the membrane permeable fluorescent indicator Fura 2-AM (Sigma-Aldrich) in combination with the Till Vision Imaging System (T.I.L.L. Photonics, Gräfelfing, Germany) coupled to an upright microscope (Axioskop 2 FS plus, Zeiss). Emitted fluorescence was collected by a charge-coupled device (CCD) camera. Cultured cells were incubated for 30 minutes at 37°C with Fura 2-AM in a standard bath solution containing 140 mM NaCl, 5 mM KCl, 2 mM CaCl_2_, 10 mM glucose and 10 mM HEPES, adjusted to pH 7.4 with NaOH.

The intracellular Ca^2+^ was imaged by exciting Fura 2-AM at 340 and 380 nm with its emission monitored in intervals of 300 ms at 510 nm. Recordings were terminated by a 50 mM KCl stimulation to ensure the viability of the recorded cells. After background subtraction, the 340/380 nm excitation ratio for Fura 2-AM was calculated, which increases as a function of the cytosolic free Ca^2+^ concentration ([Ca^2+^]_i_). To determine [Ca^2+^]_i_ a calibration measurement in the presence of 5 μM ionomycin or with a 10 mM EGTA solution free of Ca^2+^ was conducted. [Ca^2+^]_i_ was calculated according to [Ca^2+^]_i_ = β × K_D_(R ‒ R_min_)/(R_max_ ‒ R) [55] with β = F_380,max_/F_380,min_ = 3.6, K_D_ = 245 nM, R_min_ = 0.38 and R_max_ = 1.6.

### Statistics

Data were analyzed with GraphPad Prism (GraphPad Software, San Diego, CA, USA) by a two-way analysis of variance (ANOVA) and Bonferroni posttest or unpaired t-test as appropriate. All data are presented as means ± SEM and the significance level was set as *P* <0.05.

## Results

### Dorsomorphin, SB 431542, purmorphamine and FGF8 efficiently direct neural conversion and midbrain regionalization of hCBiPSCs

**Figure 1:**
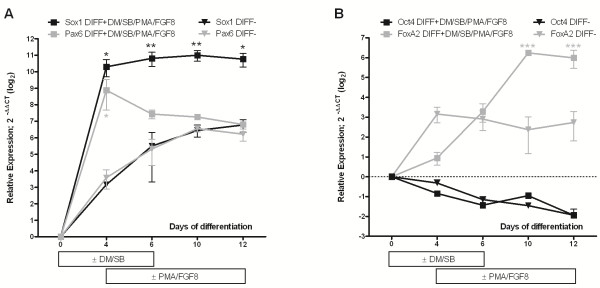
**The utilization of dorsomorphin (DM) and SB 431542 (SB) in the hCBiPSCs differentiation process significantly improved neural conversion.** Relative gene expression was measured by quantitative real-time PCR. **(A)** A marked increase in the expression of the neural stem cell markers Sox1 and Pax6 was observed during the first four days of differentiation by application of DM/SB (DIFF+) compared to cells differentiated without these molecules (DIFF-). **(B)** Downregulation of the pluripotency marker Oct4 was found as the maturation proceeded under both conditions. Midbrain regionalization, monitored by the expression of the midbrain marker FoxA2, was induced by PMA/FGF8 treatment (DIFF+) on day 4 of differentiation. A significantly higher FoxA2 level was observed in PMA/FGF8 treated cells compared to controls (DIFF-) from day 10 on. Values are calculated as means ± SEM. *P*-values (*P <0.05, **P <0.01, ***P <0.001) were determined using two-way ANOVA and Bonferroni posttest. ANOVA, analysis of variance; FGF8, fibroblast growth factor 8; hCBiPSCs, human cord blood induced pluripotent stem cells; PMA, purmorphamine; SEM, standard error of the mean.

To explore the neuronal differentiation potential of hCBiPSCs *in vitro*, we evaluated four hCBiPSC lines generated by Haase *et al*. [53] by lentiviral transduction of the four pluripotency associated transcription factors OCT4, SOX2, NANOG and LIN28 into human cord blood-derived endothelial cells. All hCBiPSC lines exhibited morphological features typical for hESCs and expressed hESC markers [53]. Based on previous studies showing a highly efficient neuronal induction by dual inhibition of SMAD signaling using the small molecules DM and SB [48,56], we first compared the efficiency of neural induction in our hCBiPSCs with and without SMAD inhibition by DM/SB during the first six days of differentiation. Already four days after the initiation of differentiation, the expression of the neural progenitor cell (NPC) markers Pax6 and Sox1 was significantly enhanced in cells treated with DM/SB (Figure 1A). Whereas the expression of Sox1 under DM/SB/PMA/FGF8 treatment was stable until 12 days of differentiation, the addition of PMA and FGF8 on day 4 reduced Pax6 expression to levels measured in untreated cells [57]. The loss of pluripotency during the maturation process monitored by Oct4 expression was comparable under both conditions (Figure 1B). After four days of differentiation, midbrain patterning was induced by addition of FGF8 and PMA, a small molecule known to be an effective substitute for sonic hedgehog [48,58]. Foxa2 expression, as an early marker for midbrain regionalization, was significantly elevated from day 10 on in cells incubated with PMA/FGF8 for eight days (Figure 1B). These data suggest a reduction of pluripotency associated with a rapid and efficient differentiation towards neuroectoderm in DM/SB-treated cells and an efficient midbrain patterning induced by PMA and FGF8.

### hCBiPSCs differentiate into DA neurons *in vitro*

**Figure 2:**
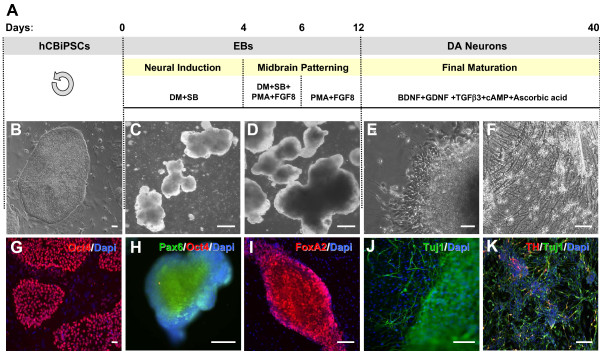
**Sequential induction of hCBiPSCs towards dopaminergic neurons. (A)** Schematic summary of the differentiation procedure. Phase-contrast images **(B-F)** and immunocytochemical stainings **(G-K)** during *in vitro* differentiation. Oct4-positive hCBiPSC colonies **(B,G)** were detached from the feeder layer and cultured in suspension as embryoid bodies (EBs) in the presence of dorsomorphin (DM) and SB 431542 (SB) for six days. During this time Oct4 expression was completely lost, whereas cells started to express the neural stem cell marker Pax6 **(H)**. On day 4, purmorphamine (PMA) and FGF8 were added to initiate regionalization. After 12 days the vast majority of EBs coexpressed the midbrain marker FoxA2 **(I)**. EBs were plated on PLO/laminin-coated cell culture dishes on day 12. Tuj1-positive neuronal cells spread out **(J)** and maturated into numerous dopaminergic (DA) neurons in the presence of BDNF, GDNF, TGFβ3, cAMP and ascorbic acid as indicated by tyrosine hydroxylase (TH)-positive cells **(K)**. Scale bars represent 100 μm. FGF8, fibroblast growth factor 8; hCBiPSC, human cord blood induced pluripotent stem cells.

**Figure 3:**
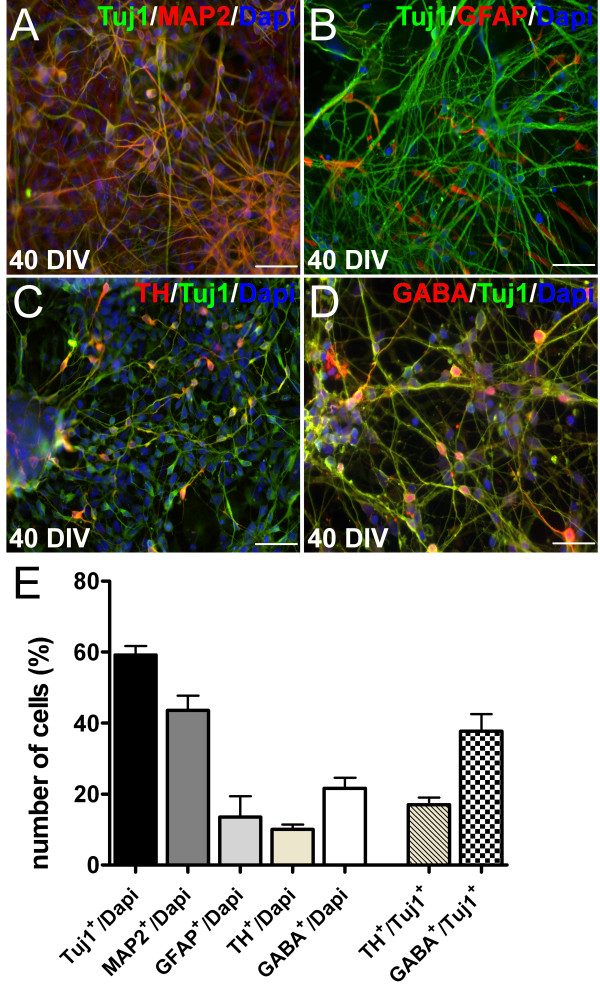
**hCBiPSCs differentiated into dopaminergic and GABAergic neurons. (A, E)** After 40 days of *in vitro* differentiation (40 DIV) almost 60% of the cells were positive for the neuronal marker beta III tubulin (Tuj1) and 40% showed reactivity against MAP2 indicating more mature neurons. A minor cell population of roughly 13% was positive for the astrocytic marker GFAP **(B, E)**. Approximately 17% of Tuj1-positive cells were double labeled with antibodies against the dopaminergic marker tyrosine hydroxylase (TH, **C**, **E**) and nearly 40% were immunopositive for GABA **(D, E)**. Scale bars represent 50 μm. Values are calculated as means ± SEM. hCBiPSC, human cord blood induced pluripotent stem cells; SEM, standard error of the mean.

Figure 2A gives a schematic overview of the differentiation protocol used in this study. Figures 2B-F demonstrate representative light microscopic images of the neuronal maturation process. During expansion hCBiPSC colonies uniformly express the pluripotency marker Oct4 (Figure 2G). After six days of *in vitro* differentiation with DM/SB, Oct4-positive cells were rarely detected, whereas the vast majority of EBs were immunopositive for the NPC marker Pax6 (Figure 2H). On day 4, PMA and FGF8 were added to suspension cultures for eight days to induce midbrain regionalization. After 12 days in suspension, EBs were positive for the midbrain marker FoxA2 (Figure 2I). After plating onto PLO/Laminin-coated cell culture dishes, cells were cultured in the presence of BDNF, GDNF, TGFβ3, dbcAMP and ascorbic acid to promote terminal differentiation. Cells growing out of the EBs displayed immunoreactivity for the neuronal marker beta III tubulin (Tuj1; Figure 2J). After almost 40 days of *in vitro* differentiation 59 ± 3% of the cells were Tuj1^+^ and 17 ± 2% coexpressed the DA marker tyrosine hydroxylase (TH; Figure 2K, Figure 3C). Furthermore, 38 ± 5% GABAergic neurons were identified among the Tuj1^+^ cells (Figure 3D). The overall cell population contained 10 ± 1% TH^+^ neurons and 22 ± 3% GABA^+^ neurons. MAP2-staining, a marker for more mature neurons, showed reactivity in 42 ± 4% of the cells, whereby nearly all of these neurons were Tuj1^+^ as well (Figure 3A). Additionally, 14 ± 6% GFAP^+^ astrocytes were present in the cultures (Figure 3B). The vast majority of cells negative for Tuj1 or GFAP expressed the neural progenitor marker Sox1 (19 ± 7%), suggesting incomplete maturation. None of the cells were positive for the oligodendrocytic markers Olig2 and O4 (data not shown).

### qRT-PCR reveals midbrain DA patterning during *in vitro* differentiation

**Figure 4:**
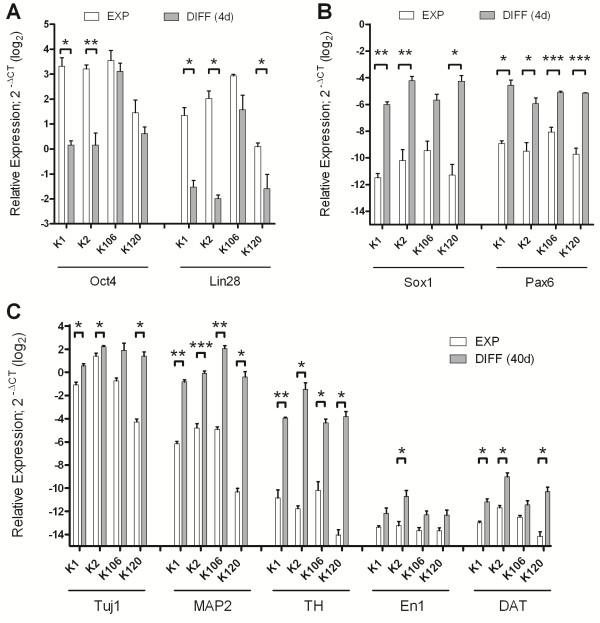
**Quantitative real-time PCR of four hCBiPSC lines.** Relative expression levels of pluripotency **(A)**, neural progenitor **(B)** and neuronal and dopaminergic markers **(C)** in four hCBiPSC clones (K1, K2, K106, K120). **(A)** The expression of the pluripotency markers Oct4 and Lin28 is significantly decreased in most cell lines during expansion whereas the neural stem cell markers Sox1 and Pax6 **(B)** are markedly upregulated in all hCBiPSC clones after differentiation with DM/SB for four days. **(C)** After 40 days of differentiation *in vitro* the relative expression of the neuronal markers Tuj1 and MAP2 is significantly enhanced in almost all clones. Furthermore, the expression levels of tyrosine hydroxylase (TH) and the dopamine transporter (DAT) which are markers for dopaminergic neurons, as well as the mesencephalic marker engrailed 1 (En1) are significantly elevated in most cell lines. Results are reported as means ± SEM. *P*-values (*P <0.05, **P <0.01, ***P <0.001) were calculated using an unpaired t-test. Abbreviations: EXP, expanded cells; DIFF, differentiated cells. DM, dorsomorphin; hCBiPSC, human cord blood induced pluripotent stem cells; SEM, standard error of the mean.

By qRT-PCR we investigated the genomic expression of several genes that are typically present in undifferentiated iPSCs, neural progenitors or differentiated DA neurons (Figure 4). Already four days after the beginning of differentiation the pluripotency marker Oct4 and Lin28, expressed in undifferentiated iPSCs, were markedly downregulated in most analyzed hCBiPSC lines (Figure 4A). Thereafter, we observed significant upregulation of transcription factors associated with the appearance of NPCs, for example, Sox1 and Pax6 (Figure 4B). These findings are consistent with the results of the immunocytochemical stainings (see above). In cultures subjected to 40 days of differentiation, we detected a significant elevation in mRNA expression for cell markers of mature midbrain DA neurons, for example, Tuj1, MAP2, TH, En1, and DAT in most of the cell lines (Figure 4C).

### Differentiated hCBiPSC-derived cells exhibit electrophysiological characteristics of functional neurons

**Figure 5:**
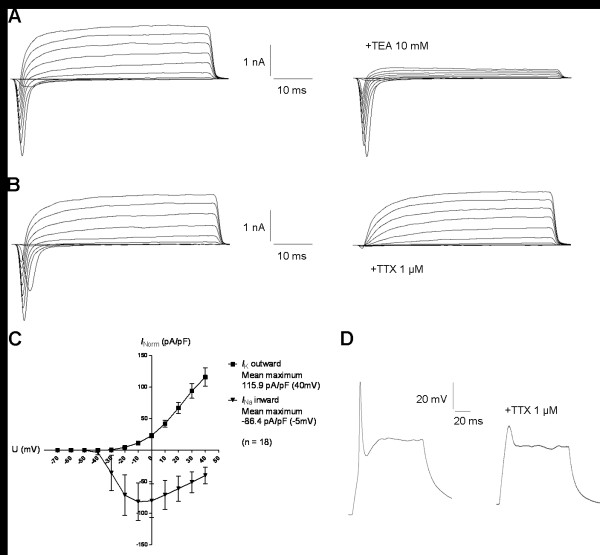
**hCBiPSCs maturated into functional neurons *in vitro*.** All functional analyses were conducted with the hCBiPSC line K2. **(A-C)** Voltage-activated sodium and potassium currents were recorded in the whole-cell voltage-clamp mode by increasing depolarizing steps of 10 mV from a holding potential of -70 to 40 mV. **(A)** Cells showed potassium outward currents (*I*_K_) that were inhibited by application of tetraethylammonium (TEA, 10 mM, n = 18). **(B)** Sodium inward currents (*I*_Na_) could be completely blocked by tetrodotoxin (TTX, 1 μM, n = 8). **(C)** The current-voltage plot indicates the activation of *I*_K_ between -30 and -20 mV. *I*_Na_ were activated between -40 and -20 mV with a current peak at -10 to 0 mV. Current amplitudes were normalized for cell capacitances and are calculated as means ± SEM. **(D)** Action potentials were elicited by a depolarizing current step of 100 pA in current-clamp mode and inhibited by application of 1 μM TTX (n = 7). Voltage-gated ion channel, passive membrane and action potential properties are summarized in Table 1. hCBiPSC, human cord blood induced pluripotent stem cells; SEM, standard error of the mean.

Fundamental neuronal properties, such as excitability and synaptic transmission, are based on the functional expression of ion channel proteins. Therefore, we examined the electrophysiological properties of hCBiPSCs-derived neurons differentiated for six weeks *in vitro*. Voltage-gated sodium and potassium channels as well as action potential properties and synaptic activity were analyzed by whole-cell patch-clamp recordings. Large outward currents were reliably induced by depolarizing voltage steps of 10 mV from a holding potential of -70 mV to 40 mV. These currents showed voltage dependence and kinetics characteristic of potassium currents and were inhibited by the potassium channel blocker TEA (10 mM) applied to the extracellular solution (Figure 5A,C). In response to depolarization, 44% of the cells generated sodium inward currents that were blocked by the sodium channel blocker TTX (1 μM; Figure 5B,C). Peak currents were normalized for cell size based on the capacitance of the cell membrane (pA/pF, Figure 5C). In current-clamp experiments 39% (n = 7/18) of the neurons fired TTX-sensitive APs with average amplitudes of 51.2 ± 8.0 mV and durations of 3.2 ± 0.7 ms (Figure 5D). A summary of the functional properties of differentiated hCBiPSCs-derived neurons can be found in Table 1.

**Table 1:** **Functional properties of hCBiPSC-K2 after differentiation for six weeks *in vitro***

**Functional properties**	**Values of differentiated hCBiPSCs (n = 18)**
Peak Na + -current	−859.1 ± 359.7 pA
Peak Na + -current/pF	−86.4 ± 31.7 pA/pF
Peak K + -current	1,034.2 ± 192.7 pA
Peak K + -current/pF	115.9 ± 20.8 pA/pF
Resting membrane potential	−34.4 ± 2.1 mV
Membrane capacitance	9.4 ± 1.1 pF
Input resistance	1,169.2 ± 389.7 MOhm
Cells with APs	7 (38.9%)
AP amplitude	51.2 ± 8.0 mV
AP duration	3.2 ± 0.7 ms
AHP amplitude	13.3 ± 3.0 mV
Time to peak AHP	10.6 ± 1.2 ms

Voltage-gated ion channel, passive membrane and action potential properties were determined by whole-cell patch clamp recordings. Data are given as means ± SEM. AP amplitude was measured from spike onset to peak, afterhyperpolarization (AHP) amplitude from peak to beginning of plateau reached during the current injection, AP duration was measured at half amplitude, and time to peak AHP from spike onset.

**Figure 6:**
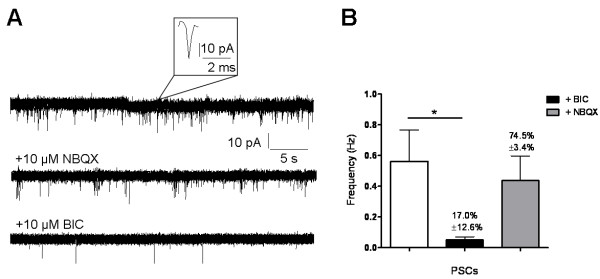
**Differentiated hCBiPSC exhibited spontaneous synaptic activity. (A)** Current traces recorded in whole-cell voltage-clamp mode from a single cell of the hCBiPSC line K2 under control conditions (top) and following application of the AMPA receptor blocker NBQX (10 μM, middle) or the GABA_A_ receptor blocker bicuculline (BIC, 10 μM, bottom). **(B)** Average frequency of spontaneous post-synaptic currents (PSCs). Application of BIC significantly reduced the PSC frequency to 17.0% (n = 5), NBQX just slightly depressed PSCs to 74.5% (n = 5). hCBiPSC, human cord blood induced pluripotent stem cells.

Another critical issue of neuronal functionality is the capability of hCBiPSC-derived neurons to form synaptic connections. This was explored by measuring spontaneous post-synaptic currents (PSCs) due to action potential-independent transmitter release, using the whole-cell voltage-clamp configuration at a holding potential of -70 mV. Spontaneous PSCs were measured in 40% of the cells showing an average frequency of 0.6 ± 0.2 Hz, which was reduced to 17.0 ± 12.6% by application of the GABA_A_ receptor blocker bicuculline (BIC) (n = 5, Figure 6A and B). Inhibiting glutamatergic input by NBQX decreased the PSC frequency to only 74.5 ± 3.4%, indicating a predominantly GABAergic synaptic input. The mean amplitudes of spontaneous PSCs were 21.4 ± 3.7 pA.

These data show that hCBiPSCs can give rise to functional neurons acquiring mature electrical properties and spontaneously active synaptic contacts during their differentiation *in vitro*.

### Neurotransmitters induce increases in [Ca^2+^]_i_

**Figure 7:**
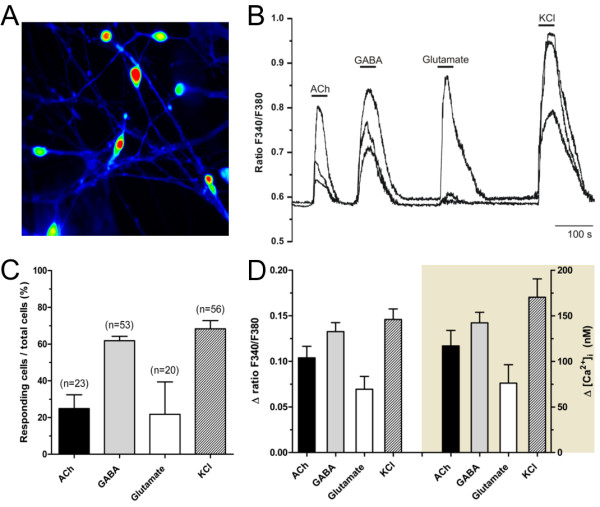
**Differentiated hCBiPSCs showed KCl- and neurotransmitter-mediated Ca**^**2+ **^**signaling. (A)** hCBiPSC-K2 were loaded with Fura-2 after four weeks of differentiation *in vitro*. **(B)** Transient cytosolic Ca^2+^ changes of three representative cells induced by bath application of acetylcholine (ACh, 100 μM), GABA (100 μM), glutamate (50 μM) and KCl (50 mM). Intracellular calcium concentrations are presented as ratios of the fluorescence signals obtained at 340 and 380 nm (F340/F380). **(C)** Fractions of cells responding to ACh (25%), GABA (62%), glutamate (22%) and KCl (68%) obtained from experiments as shown in **(B)**. **(D)** Summary of cytosolic Ca^2+^ response amplitudes given as ratio F340/F380 (left) and intracellular calcium concentration ([Ca^2+^]_i_, right) both normalized to the basal calcium level of the cells. Basal intracellular Ca^2+^ level was 100 ± 8 nM (n = 57 cells). Note, GABA induced depolarizing effects in most investigated cells. All data are given as mean ± SEM. hCBiPSC, human cord blood induced pluripotent stem cells; SEM, standard error of the mean.

The expression of functional ligand-gated channels in hCBiPSC-derived neurons was examined by measuring intracellular Ca^2+^ changes upon application of the neurotransmitters acetylcholine (ACh), GABA and glutamate in fura-2 loaded cells. Figure 7A shows typical neurons used during these experiments. For quantification of the intracellular Ca^2+^ concentration ([Ca^2+^]_i_) we performed calibration measurements. The basal Ca^2+^ level was R_F340/F380_ = 0.58, which corresponds to a basal [Ca^2+^]_i_ of 100 ± 8 nM (n = 57 cells). Figure 7B illustrates Ca^2+^ traces of three representative cells upon stimulation by ACh, GABA and glutamate as well as the depolarizing agent KCl. The excitatory neurotransmitter ACh and glutamate induced an increase in intracellular Ca^2+^ in 25 ± 8% and 22 ± 18% of the cells, respectively (Figure 7C). GABA, as the most prominent inhibitory neurotransmitter in the adult central nervous system (CNS), induced a Ca^2+^ response in 62 ± 2% of the cells suggesting depolarizing excitatory GABA-effects in most neurons. Ca^2+^ responses to KCl application were shown by 68 ± 4% of the cells indicating the neuronal population. On average, the application of ACh led to an increase of the fluorescent signal of R_F340/F380_ = 0.10 ± 0.01, which correlates to an increase in cytosolic Ca^2+^ of 117 ± 17 nM (Figure 7D). GABA induced a [Ca^2+^]_i_ rise of 142 ± 12 nM and glutamate application resulted in a [Ca^2+^]_i_ boost of 76 ± 20 nM. KCl as a depolarizing agent leading to the activation of voltage-dependent calcium channels induced the highest Ca^2+^ response of 170 ± 20 nM and indicated the viability of cells at the end of each measurement. Our data show that hCBiPSC-derived neurons develop functional ACh, GABA and glutamate receptors during differentiation *in vitro*.

## Discussion

We demonstrated the ability of iPSCs generated from human cord blood endothelial cells [53] to be directed to a neuronal cell fate similar to hiPSC lines obtained from adult somatic cell sources. The small molecules DM and SB, that have been shown to rapidly induce neuralization in hESCs and hiPSCs by dual inhibition of TGF-β/BMP signaling [48,51,56,59,60], were able to enhance neural conversion efficiency in our hCBiPSC cultures as well. DM, an antagonist of the BMP pathway, selectively blocks the BMP type I receptors ALK2, ALK3 and ALK6, and thereby inhibits downstream SMAD1/5/8 signaling [48,50,61]. SB is a selective blocker of the TGF-β/activin pathway. It targets activin receptor-like kinase receptors ALK4, ALK5 and ALK7, leading to inhibition of SMAD2/3 signaling [49,56,59,60,62,63]. The synergistic block of both signaling cascades retards differentiation towards endoderm, mesoderm and trophoectoderm and facilitates neuroectodermal differentiation as suggested by gene expression analysis and immunostainings for neural precursor markers [48,51].

The percentage of Tuj1-positive neurons achieved in this study by dual SMAD inhibition was approximately 60%. To our knowledge, there are only two studies using the same combination of small molecules (DM/SB) for neural conversion of hiPSCs from adult skin fibroblasts [48,56]. Mak and colleagues [48] reported 86% Tuj1^+^ neurons and 9% TH^+^/Tuj1^+^ cells after enrichment of NPCs via microbeads. We achieved a nearly twofold amount of TH^+^/Tuj^+^ cells (17%). Further publications performing SMAD inhibition with noggin, another BMP antagonist, instead of DM or with additional stimulation of WNT signaling [13,51,64,65] obtained neuronal differentiation efficiencies of fibroblast-derived hiPSCs ranging from 17% to 70% Tuj1^+^ cells. The newest approach skipping the iPSC intermediate stage and directly reprogramming human fibroblasts to neurons yielded only 10% to 16% Tuj1^+^ cells [66,67]. In relation to those results, our culturing strategy resulted in more efficient neurogenesis. The reasons for the heterogeneity in the differentiation capacity of different iPSC lines are not fully understood yet. There are slight variations of gene expression possibly due to differential promoter binding by the reprogramming factors [12,68,69] or epigenetical reasons [70]. Hirose *et al*. [71] discussed that the initial culture conditions for maintaining the undifferentiated state strongly influence the differentiation propensity. The percentage of TH^+^ DA neurons was consistent with most of the previous results from other research groups, reporting between 3% to 30% TH^+^ cells per total cells from fibroblast-derived hiPSCs or directly reprogrammed human fibroblasts [6,10,12,43,65-67]. Higher percentages of TH^+^/Tuj1^+^ neurons (35% to 65%) could only be achieved by overexpression of developmental transcription factor, for example, LMX1A either alone or in combination with ASCL1 and NURR1, or by co-culturing on stromal feeder cells [9,64,72,73].

It is even more important that neurons generated from hiPSCs are functional rather than just exhibiting a neuronal morphology and gene expression pattern. The number of studies dealing with functional properties of neurons generated from hiPSCs is currently quite limited [12-14,51,70]. While most studies show a few neuronal features, we conducted a detailed analysis on voltage- and ligand-gated ion channels. Our data demonstrate the maturation of neuronal function in hCBiPSCs-derived cells after *in vitro* differentiation. Previous approaches to characterize fibroblast-derived hiPSCs elucidated that differentiated cells possessed voltage-gated current amplitudes of *I*_Na_ -195 pA/pF and *I*_K_ 208 pA/pF and were able to generate multiple APs [12]. The currents we measured displayed somewhat smaller mean amplitudes of *I*_Na_ -86 pA/pF and *I*_K_ 116pA/pF which were comparable to those seen in neurons directly reprogrammed from human fibroblasts [66]. Also, trains of APs or spontaneous firing as reported from some research groups [13,74] were absent in our hCBiPSCs-derived neurons suggesting a yet incomplete neuronal maturation after 40 days of differentiation. This is in line with the recorded resting membrane potential of -34 mV, which is slightly below the values (-39 to -58 mV) that had been reported for hESC-derived neurons or neurons generated directly from human fibroblasts [44,66,67,74]. The percentage of cells with inducible APs strongly varies among several publications. We recorded nearly 40% neurons with APs, Stover *et al*. [14] observed just 14%, whereas Zeng and colleagues [12] obtained 75% firing cells. However, we were not able to yield as high percentages of cells (50% to 100%) with inducible APs as studies using hESCs [74] or methods to directly convert fibroblast to DA neurons (71% to 83%; [66,67]). The AP amplitudes of 51 mV on the other hand were similar to previous observations for hiPSC-derived neurons (50 mV [12]) and within the range of studies with hESC-derived nerve cells (35 to 50 mV [74]; 32 mV [44]; 74 to 84 mV [60]) and direct reprogrammed fibroblasts (approximately 45 mV [67]; 78 mV [66]). AP durations (3 ms) were also in the reported range of 1 to 7 ms [44,60,66,67,74]. Whether prolonged *in vitro* differentiation can give rise to fully mature hCBiPSC-derived neurons or if *in vivo* maturation will be required, as shown by Wernig *et al*. [75], remains to be investigated.

Measuring spontaneous PSCs as a sign of synaptic connectivity in neuronal systems revealed that 40% of differentiated cells exhibited spontaneous activity. This is consistent with the work of Johnson *et al*. [74], observing maximal 50% of neurons with PSCs among differentiating hESCs regardless of the maturation period. Schaarschmidt *et al*. [76] reported 94% of neurons differentiated from human fetal NPCs to receive synaptic input. Interestingly, Johnson *et al*. [74] found that the onset of synaptic activity is associated with the outgrowth of astrocytes in their cultures. Likewise, multiple other studies have shown that co-cultivating neurons with astrocytes enhances synaptogenesis [77-79]. Thus, the disparity between the results could possibly be attributed to the number of astrocytes in the cultures. While we had 14% GFAP^+^ cells after differentiation, Schaarschmidt and colleagues [76] reported more than 30%.

To our knowledge, we are the first group investigating ligand-gated ion channels in differentiated neurons from hiPSCs at all. We found that Ca^2+^ transients in differentiated hCBiPSCs rose in a less pronounced fashion and in a fewer percentage of cells (22%) when glutamate receptors were stimulated, compared to studies with differentiated human fetal NPCs (>95%) [80]. On the other hand, during application of GABA more cells (62%) responded with higher Ca^2+^ amplitudes in comparison with differentiated fetal NPCs (48%) [81]. The calcium imaging results confirm our electrophysiological data indicating a not yet fully mature neuronal phenotype. This assumption is supported by the excitatory action of GABA in differentiated hCBiPSCs. The ability of GABA to depolarize cells depends on the intracellular Cl^−^ concentration. If the Cl^−^ importer NKCC1 is expressed more pronounced than the Cl^−^ exporter KCC2, as it is in the prenatal state of development, the intracellular Cl^−^ concentration is high and GABA has a depolarizing effect, because of a Cl^−^ efflux [82,83]. Nevertheless, we were able to show that hCBiPSC-derived neurons exhibit functional ligand-gated ion channels during their maturation *in vitro*.

Given their easy accessibility and low immunogenicity, the interest in using iPSCs for regenerative cell therapy is high. Animal studies have shown that iPSC-derived neurons survive and integrate into the host brain and are able to reduce motor symptoms in parkinsonian animal models [6,9,10,64,75]. However, several critical issues such as graft survival and overgrowth or tumor formation remain obstacles to further preclinical studies. It will have to be determined if the usage of juvenescent rather than adult human cells for the derivation of iPSCs may help to overcome these problems.

## Conclusions

Our data indicate the successful and highly efficient *in vitro* generation of hCBiPSC-derived neurons including dopaminergic cells. We provide a detailed functional analysis of voltage- and ligand-gated ion channels which is a prerequisite for clinical applications. Our hCBiPSC-derived neurons exhibit essential functional properties and may serve as a juvenescent cell source for the development of novel regenerative treatment strategies.

## Abbreviations

(D)MEM: (Dulbecco’s) modified Eagle’s medium; Ach: acetylcholine; AHP: afterhyperpolarization; AP: action potential; BIC: bicuculline; BMP: bone morphogenetic protein; BSA: bovine serum albumin; DA: dopamine; DA: dopamine; DAT: dopamine transporter; DM: dorsomorphin; EBs: embryoid bodies; FGF: fibroblast growth factor; hCBiPSCs: human cord blood endothelial cell-derived induced pluripotent stem cells; hESCs: human embryonic stem cells; NPCs: neuronal progenitor cells; PBS: phosphate-buffered saline; PMA: purmorphamine; PSC: postsynaptic currents; SEM: standard error of the mean; TEA: tetraethylammonium chloride; TGF-β: transforming growth factor-β; TH: tyrosine hydroxylase; TTX: tetrodotoxin.

## Competing interests

The authors declare that they have no competing interests.

## Authors’ contributions

NS: *in vitro* experiments, data analysis and interpretation, manuscript writing and final approval of the manuscript. AH and UM: generation of induced pluripotent stem cells and establishment of their cultivation, analysis and interpretation of data, critical revision and final approval of the manuscript. MN and AL: contribution to electrophysiological and calcium imaging analysis and data interpretation, critical revision and final approval of the manuscript. RD: conception and design of the experiments, critical revision and final approval of the manuscript. FW: conception and design of the study, data analysis and interpretation, drafting and final approval of the manuscript. All authors agree to be accountable for all aspects of the work in ensuring that questions related to the accuracy or integrity of any part of the work are appropriately investigated and resolved. All authors read and approved the final manuscript.

## Supplementary Material

Additional file 1: Figure S1 Validation of amplicon sizes. The correct sizes of the amplification products were determined by agarose gel electrophoresis. Product sizes are indicated below the image. DNA ladder reached from 25 to 766 base pairs (bp). Click here for file

Additional file 2: Table S1 Oligonucleotides for quantitative real-time PCR analysis of pluripotency and differentiation marker expression in hCBiPSCs. Melting temperatures and sequences of oligonucleotides as well as sizes of amplification products in base pairs are given for each investigated marker gene. Click here for file
