# Supplementary figures and images for: Functional differentiation of midbrain neurons from human cord blood-derived induced pluripotent stem cells
Source: Stem Cell Res Ther. 2014 Mar 17;5(2):35. doi: 10.1186/scrt423 (PMC4055096; doi:10.1186/scrt423)

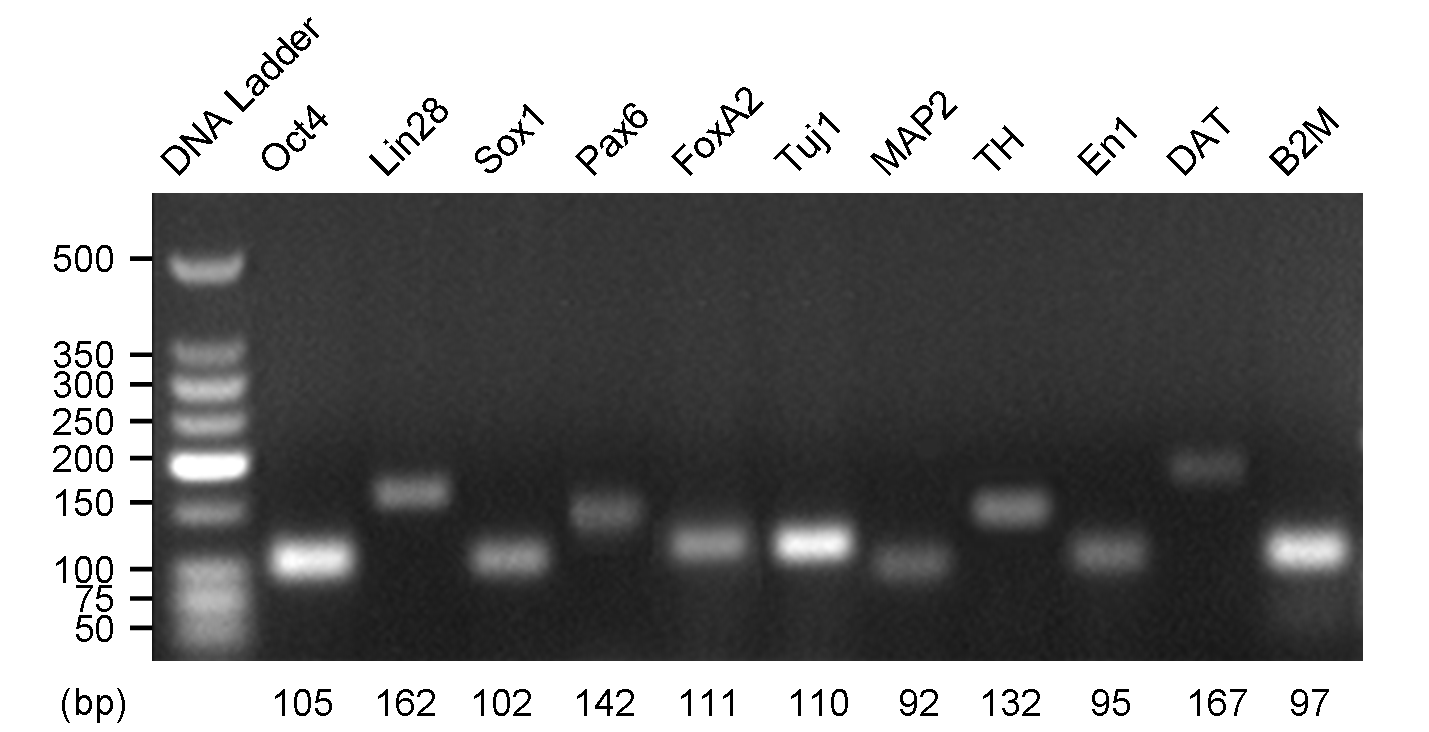

Supplement: Additional file 1: Figure S1 — Validation of amplicon sizes. The correct sizes of the amplification products were determined by agarose gel electrophoresis. Product sizes are indicated below the image. DNA ladder reached from 25 to 766 base pairs (bp). [file scrt423-S1.tiff]
