# Supplementary material for: Functional differentiation of midbrain neurons from human cord blood-derived induced pluripotent stem cells
Source: Stem Cell Res Ther. 2014 Mar 17;5(2):35. doi: 10.1186/scrt423 (PMC4055096; doi:10.1186/scrt423)
Supplement: Additional file 2: Table S1 — Oligonucleotides for quantitative real-time PCR analysis of pluripotency and differentiation marker expression in hCBiPSCs. Melting temperatures and sequences of oligonucleotides as well as sizes of amplification products in base pairs are given for each investigated marker gene. [file scrt423-S2.doc]

**Additional file 2: Table S1** Oligonucleotides for quantitative real-time PCR analysis of pluripotency and differentiation marker expression in hCBiPSCs. Melting temperatures and sequences of oligonucleotides as well as sizes of amplification products in base pairs are given for each investigated marker gene.

| Gene | Tm [°C] | Sequence (5’-3’; forward, reverse) | Product size (bp) |
| --- | --- | --- | --- |
| Oct4 | 64.4 | CACCCTGGGGGTTCTATTTGGGAA, | 105 |
|  | 63.7 | AGCAAGGGCCGCAGCTTACAC |  |
| Lin28 | 61.4 | TTGAGGAGCAGGCAGAGTGG, | 162 |
|  | 57.3 | TGCATTTGGACAGAGCATGG |  |
| Sox1 | 62.7 | GGCTTTTGTACAGACGTTCCCACA, | 102 |
|  | 67.6 | GCCAGGGTCTCCGGGGAAGG |  |
| Pax6 | 63.0 | TGCTGGAGGATGATGACAGAGGAAT, | 142 |
|  | 62.7 | TGCTGCTGTTGTTGCTTGAAGACC |  |
| Foxa2 | 61.0 | CCATGCACTCGGCTTCCAG, | 111 |
|  | 59.4 | TGTTGCTCACGGAGGAGTAG |  |
| Tuj1 | 64.2 | AGTGATGAGCATGGCATCGACCC, | 110 |
|  | 64.2 | GGCACGTACTTGTGAGAAGAGGC |  |
| MAP2 | 64.6 | CAGGCAAAGGACAAAGTCTCTGACG, | 92 |
|  | 67.7 | CGCCGAGGAGGGAGAATGGAGG |  |
| TH | 61.4 | AGCCCTACCAAGACCAGACG, | 132 |
|  | 58.8 | GCGTGTACGGGTCGAACTT |  |
| En1 | 65.5 | CACGTGCGGCCCTGACTCAC, | 95 |
|  | 64.6 | CGGATCTTTGCACAAACTCTCTCGC |  |
| DAT | 55.3 | TGCGTGCCACATCAATAACA, | 167 |
|  | 58.1 | AACATCCTTCACTCAGTATTGCTAA |  |
| B2M | 61.8 | TGCCTGCCGTGTGAACCATGT | 97 |
|  | 62.7 | TGCGGCATCTTCAAACCTCCATGA |  |
